# Spatial Distribution of Intracranial Vessel Wall Enhancement in Hypertension and Primary Angiitis of the CNS

**DOI:** 10.1038/s41598-019-55634-5

**Published:** 2019-12-17

**Authors:** Jae W. Song, Haochang Shou, Emmanuel C. Obusez, Scott B. Raymond, Samuel D. Rafla, G. Abbas Kharal, Pamela W. Schaefer, Javier M. Romero

**Affiliations:** 1 Department of Radiology, Division of Neuroradiology, Hospital of the University of Pennsylvania, Philadelphia, USA; 2 Department of Biostatistics, Epidemiology and Informatics, University of Pennsylvania Perelman School of Medicine, Philadelphia, USA; 3 Division of Neuroradiology, Massachusetts General Hospital, Harvard Medical School, Massachusetts, USA; 4 Department of Neurology, Massachusetts General Hospital, Harvard Medical School, Massachusetts, USA; 5 Present Address: Department of Radiology, University of Vermont College of Medicine, Burlington, USA; 6 Present Address: Department of Neurology, Cleveland Clinic, Ohio, USA

**Keywords:** Diagnostic markers, Stroke

## Abstract

We hypothesized a difference in the spatial distribution of intracranial vessel wall enhancement between CNS vasculitis and risk factors for intracranial atherosclerotic disease (ICAD). Fifty-five vessel wall MR imaging (VWI) exams were included in this retrospective observational study. Intracranial arteries were evaluated for vessel wall enhancement by branching pattern (e.g., primary, secondary, and tertiary segments). Demographic and laboratory data as well as ICAD risk factors, including a diagnosis of hypertension, were collected. A diagnosis of primary angiitis of the CNS (PACNS) was confirmed by biopsy or clinical assessment by a stroke neurologist. Univariate and multivariate Poisson regression models were fit for the outcomes. In multivariate analyses, hypertension showed significant associations with primary (β = 1.31, 95% CI 0.78–1.88, *p* < 0.0001) and secondary (β = 1.15, 95% CI 0.29–2.18, *p* = 0.05) segments, contrasting with PACNS which showed a distal spatial distribution with significant associations with secondary (β = 0.77, 95% CI 0.14–1.39, *p* = 0.05) and tertiary (β = 1.34, 95% CI 0.68–2.01, *p* < 0.0001) segments. Our results suggest the spatial distribution of vessel wall enhancement is an important consideration when interpreting VWI exams, particularly in patients with a comorbid diagnosis of hypertension. Given the global prevalence of hypertension, these results are impactful and may improve image interpretation of VWI in stroke patients.

## Introduction

Vessel wall MR imaging (VWI) is becoming more frequently used to assess intracranial vasculopathies. Vessel wall morphology and enhancement provides additional information not appreciable by conventional luminal techniques. Common vessel wall imaging characteristics include circumferential and eccentric wall enhancement patterns. While most studies suggest that an eccentric wall enhancement pattern reflects intracranial atherosclerotic disease (ICAD) and a circumferential wall enhancement pattern is more likely to represent an infectious or inflammatory vasculitis, an overlap of these enhancement patterns exist^1,2^. In ICAD, circumferential vessel wall enhancement may be related to neovascularization of the vessel wall as it remodels^3,4^. In inflammatory vasculitis, circumferential vessel wall enhancement has been described in primary angiitis of the CNS (PACNS) related to an inflammatory infiltrate involving the endothelial lining^5–7^. This overlap in imaging findings of a vessel segment can confound VWI interpretation when assessing for stroke etiology.

One of the strongest risk factors for ICAD is hypertension^8^. The World Health Organization reports an estimated 40% of the world population is affected by hypertension^7^. The prevalence of hypertension increases with age^9^, which raises concerns given an aging population. Moreover, hypertension is especially prevalent in stroke patients^10^, which is the predominant patient population for which VWI is performed. Thus, understanding the effects of hypertension on intracranial arterial wall changes, such as enhancement features, is important and may provide further insight when interpreting VWI exams in stroke patients.

We hypothesized that the spatial distribution of intracranial arterial wall enhancement may be affected by the different types of vasculopathies. PACNS, for instance, is considered a small vessel vasculitis, predominantly involving the small caliber vessels^11^. This spatial distribution may contrast with hypertension and other cardiovascular risk factors of ICAD, which typically involve larger caliber proximal vessels^12^. Given the possible overlapping imaging findings on VWI, our aim was to compare the spatial distribution of vessel wall enhancement in patients with PACNS compared to hypertension and other risk factors of ICAD.

## Results

### Patient and imaging characteristics

One hundred consecutive brain magnetic resonance imaging (MRI) exams with VWI were reviewed. Of these, the following numbers of cases were excluded: 19 follow-up exams; 12 motion-degraded exams; 1 motion-degraded, follow-up exam; 7 exams of patients under 18 years old; 3 cases of moyamoya syndrome/disease, 2 cases of reversible cerebral vasoconstriction syndrome, and 1 case of dissection. Fifty-five brain MR exams with VWI were included in the analysis for a 1,265 per-segment analysis. Table 1 reports the demographic characteristics. No significant differences were identified in demographic or medical comorbidity characteristics between patients with PACNS versus hypertension. Among the 8 cases diagnosed with PACNS, 3 were biopsy-proven and 5 were clinically diagnosed noting clinical improvement with treatment during follow-up. Among the 8 cases with a diagnosis of PACNS, 75% (n = 6) had a comorbid diagnosis of hypertension. There was 82% agreement for scoring the vessels with an inter-rater reliability of к = 0.39 (95% CI 0.33–0.45, *p* < 0.03; Supplemental Table 1). Supplemental Table 2 shows the median number of enhancing vessel segments among all 55 cases and among patients with hypertension, PACNS, or both.

**Table 1:** Demographics.

	All patients (n = 55)	PACNS (n = 8)	Hypertension (n = 37)	*p*-values
Age (mean (SD))	57 years (range: 22–91)	51.4 (18.52)	62.9 (12.74)	0.42
Sex				0.13
Male (n, %)	65%, (n = 36)	7 (87.5%)	24 (64.9%)	
Ethnicity (n, %)				0.74
White	71% (n = 39)	5 (62.5%)	28 (75.7%)	
Asian	5% (n = 3)	1 (12.5%)	0 (0%)	
Hispanic	2% (n = 1)	0 (0%)	1 (2.7%)	
Black	9% (n = 5)	1(12.5%)	4 (10.8%)	
Nonhispanic/Other/Unknown	13% (n = 7)	1(12.5%)	4 (10.8%)	
**Risk Factors**				
Diabetes Mellitus	67% (n = 37)	5 (62.5%)	17 (45.9%)	0.12
Body Mass Index	29.3 kg/m^2^ (range: 19–57)	29.9 (6.60)	30.5 (6.71)	0.68
Smoker^*^	45% (n = 25)	3 (42.9%)	18 (50%)	0.63
Hyperlipidemia	38% (n = 21)	3 (37.5%)	19 (51.4%)	0.94

^*^One subject had missing information about smoking history.

Abbreviations: SD, standard deviation.

### Regression analysis

Univariate analyses using Poisson regression models with the total number of enhancing vessel segments as the dependent variable were carried out to screen covariates to be included in the multivariate models (Table 2). The covariates included: age, sex, body mass index, hypertension, diabetes mellitus, hyperlipidemia, and smoking history^13^. A confirmed diagnosis of PACNS was also included. Among these covariates, age (β = 0.02, 95% CI 0.01–0.03, *p* < 0.001), hypertension (β = 1.33, 95% CI 0.98–1.70, *p* < 0.0001), diabetes mellitus (β = 0.75, 95% CI 0.53–0.98, *p* < 0.0001), hyperlipidemia (β = 0.50, 95% CI 0.27–0.73, *p* < 0.0001) and PACNS (β = 0.87, 95% CI 0.62–1.12, *p* < 0.0001) were significantly associated with vessel wall enhancement.

**Table 2:** Univariate Poisson Regression.

Number of enhancing vessel segments	βvalue (95% CI)	*p* value
Age	0.02 (0.01–0.03)	<0.0001*
Sex^†^	0.59 (0.33–0.87)	<0.0001*
Body Mass Index	0.009 (−0.006–0.02)	0.25
Hypertension^††^	1.33 (0.98–1.70)	<0.0001*
Diabetes Mellitus^‡^	0.75 (0.53–0.98)	<0.0001*
Hyperlipidemia^§^	0.50 (0.27–0.73)	<0.0001*
Smoking History	−0.10 (−0.33–0.14)	0.43
Primary angiitis of the CNS^||^	0.87 (0.62–1.12)	<0.0001*

Abbreviations: CI, confidence interval.

*p ≤ 0.05.

^†^1 = Male, 0 = Female.

^††^1 = Hypertension, 0 = No hypertension.

^‡^1 = Diabetes mellitus, 0 = No Diabetes mellitus.

^§^1 = Hyperlipidemia, 0 = No hyperlipidemia.

^||^1 = PACNS, 0 = No PACNS.

Multivariate analyses showed that when taking into account age, sex, hypertension, diabetes mellitus, hyperlipidemia, and PACNS for all vessel segments (primary, secondary, and tertiary), both hypertension and PACNS were significantly associated with wall enhancement (hypertension, β = 1.04, 95% CI 0.64–1.46, *p* < 0.0001; PACNS, β = 0.65, 95% CI 0.36–0.95, *p* < 0.0001; Table 3).

**Table 3:** Multivariate Poisson Regression.

	βvalue (95% CI)	*p* value	Adjusted *p* value
**Model 1: All intracranial segments**			
Age	0.01 (0, 0.018)	0.05	0.14
Sex	0.42 (0.12, 0.72)	0.006	0.02
Hypertension^†^	1.04 (0.64, 1.46)	<0.0001	<0.0001
Diabetes Mellitus^‡^	0.14 (−0.13, 0.41)	0.30	0.46
Hyperlipidemia§	0.001 (−0.26, 0.26)	0.99	0.99
Primary angiitis of the CNS^||^	0.65 (0.36, 0.95)	<0.0001	<0.0001
**Model 2: Primary**^**#**^			
Age	0.01 (−0.002, 0.02)	0.10	0.19
Sex	0.56 (0.18, 0.94)	0.004	0.02
Hypertension	1.31 (0.78, 1.88)	<0.0001	<0.0001
Diabetes Mellitus	0.16 (−0.18, 0.51)	0.35	0.49
Hyperlipidemia	−0.28 (−0.61, 0.05)	0.09	0.18
Primary angiitis of the CNS	0.37 (−0.03, 0.76)	0.06	0.14
Model 3: Secondary^**^			
Age	0.01 (−0.005, 0.03)	0.14	0.24
Sex	0.18 (−0.41, 0.81)	0.56	0.67
Hypertension	1.15 (0.29, 2.18)	0.02	0.05
Diabetes Mellitus	0.06 (−0.51, 0.62)	0.83	0.91
Hyperlipidemia	0.32 (−0.21, 0.87)	0.24	0.39
Primary angiitis of the CNS	0.77 (0.14, 1.39)	0.01	0.05
**Model 4: Tertiary**^**††**^			
Age	−0.002 (−0.02, 0.02)	0.87	0.91
Sex	0.31 (−0.44, 1.14)	0.44	0.59
Hypertension	0.10 (−0.79, 1.03)	0.83	0.91
Diabetes Mellitus	0.26 (−0.49, 1.01)	0.50	0.63
Hyperlipidemia	0.63 (−0.03, 1.33)	0.07	0.14
Primary angiitis of the CNS	1.34 (0.68, 2.01)	<0.0001	<0.0001

Abbreviations: CI, confidence interval.

^†^1 = Hypertension, 0 = No hypertension.

^‡^1 = Diabetes mellitus, 0 = No Diabetes mellitus.

^§^1 = Hyperlipidemia, 0 = No hyperlipidemia.

^||^1 = PACNS, 0 = No PACNS.

^#^Basilar artery and bilateral A1, M1, P1, V4, ICA terminus (11 segments).

^**^Bilateral A2, M2, P2 (6 segments).

^††^Bilateral A3, M3, P3 (6 segments).

### Assessing spatial distribution

**Figure 1:**
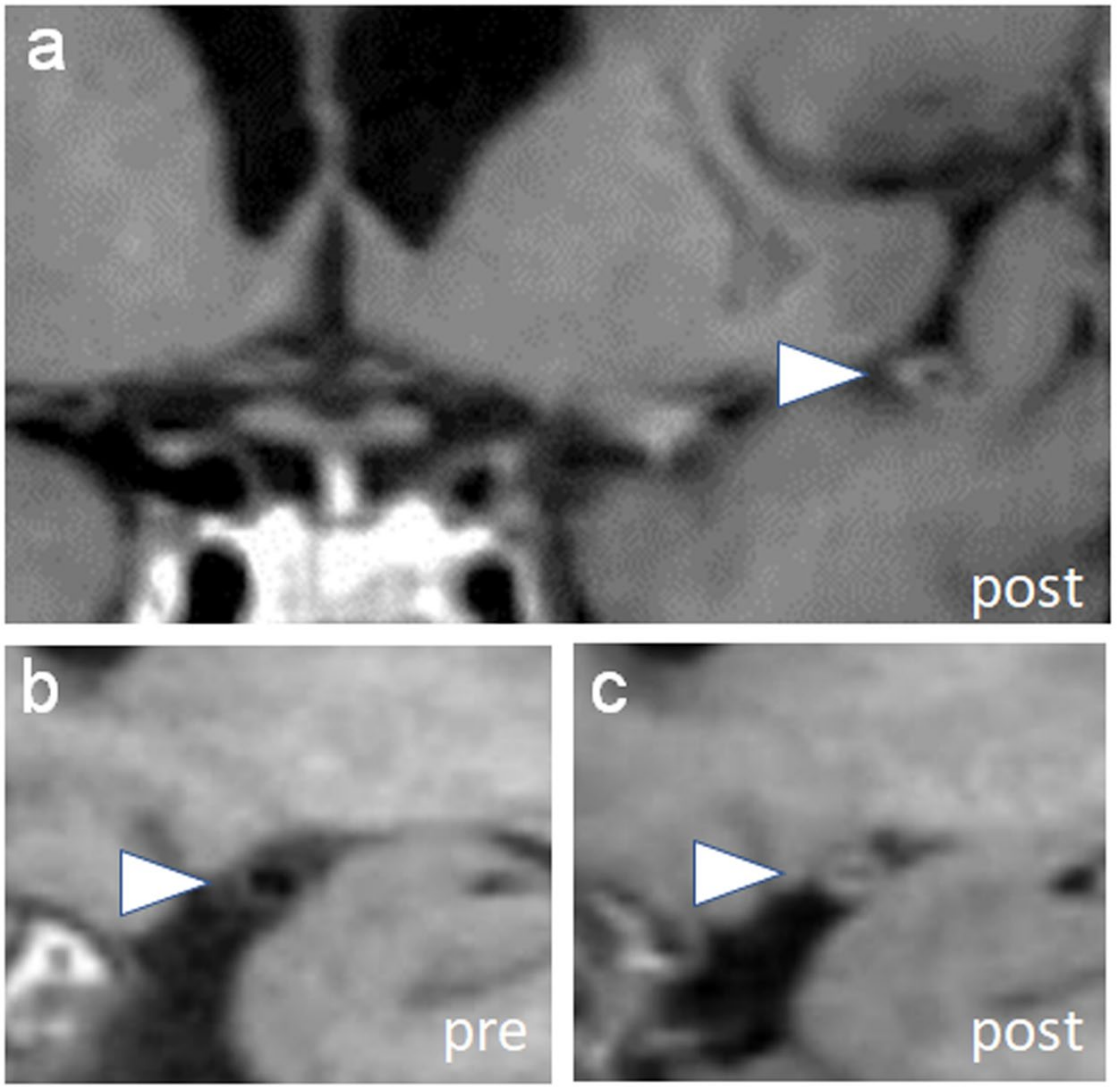
Vessel wall enhancement in primary segments. (**a**) Vessel wall imaging in a patient with primary angiitis of the CNS and comorbid diagnosis of hypertension shows circumferential wall thickening of a left distal M1 middle cerebral artery (arrowhead, MCA). (**b**,**c**) Pre and post-contrast images through the M1 MCA shows circumferential enhancement (arrowheads).

**Figure 2:**
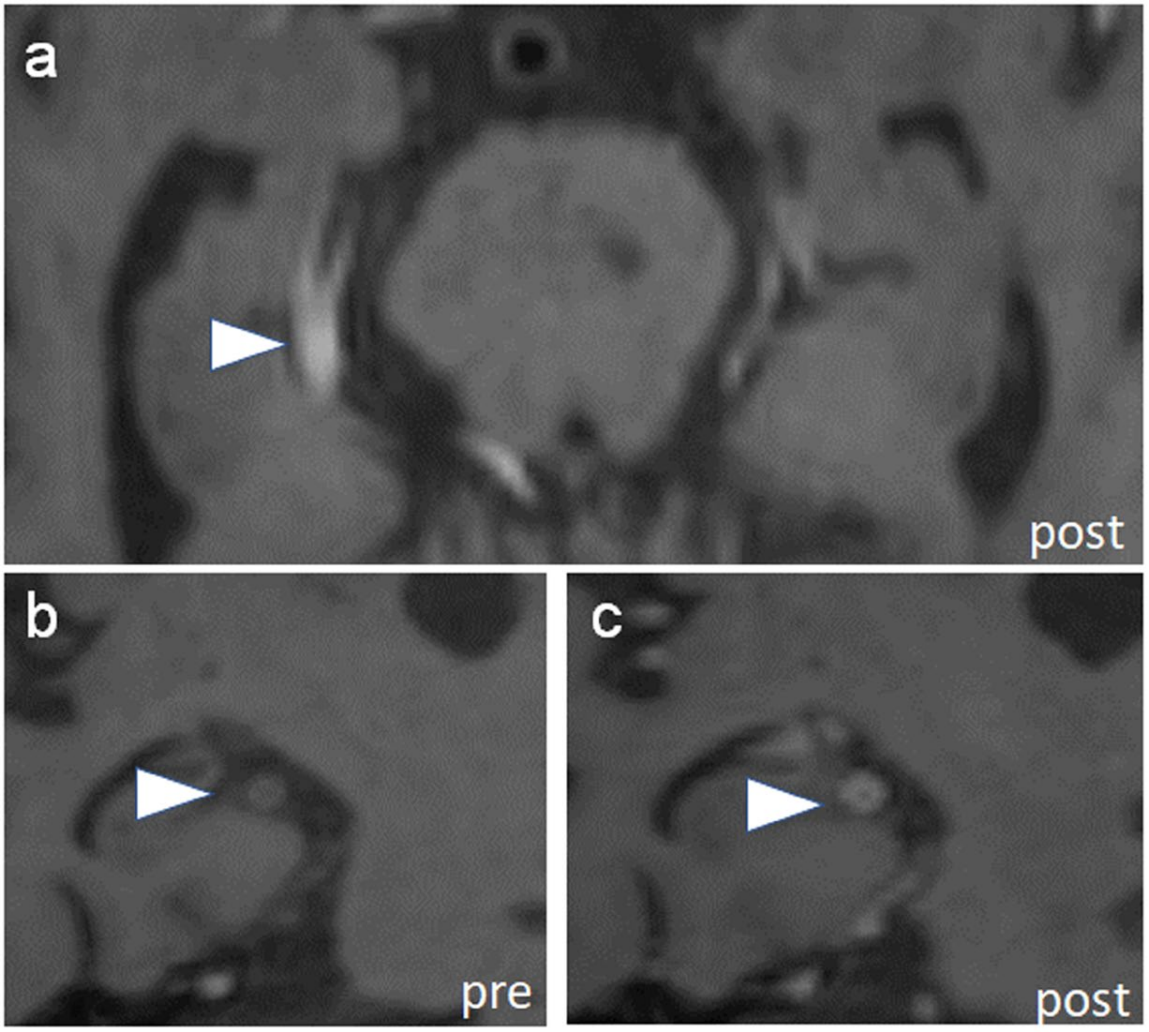
Vessel wall enhancement in secondary segments. (**a**) Vessel wall imaging in a patient with primary angiitis of the CNS and comorbid diagnosis of hypertension shows circumferential wall thickening in the right P2 posterior cerebral artery (PCA, arrowhead). (**b**,**c**) Pre and post-contrast orthogonal images through the P2 PCA shows circumferential enhancement (arrowheads).

**Figure 3:**
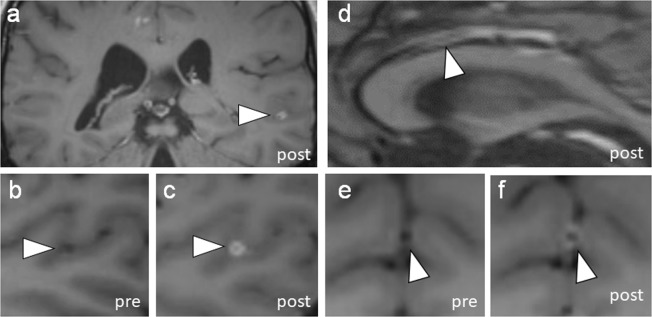
Vessel wall enhancement in tertiary segments. (**a**) In a patient with primary angiitis of the CNS, a distal M3 middle cerebral artery shows circumferential wall enhancement and thickening (MCA, arrowhead). (**b**,**c**) Insets of the left M3 MCA in orthogonal planes show pre- and post-contrast imaging to show enhancement. (**d**) Vessel wall imaging in a different patient with primary angiitis of the CNS also shows wall thickening and enhancement of a distal A3 anterior cerebral artery segment (ACA, arrowhead). (**e**,**f**) Insets show pre and post-contrast images of the enhancing vessel segment.

To evaluate for spatial distribution, three subgroup analyses were performed fitting models with the total number of enhancing primary, secondary, and tertiary segments (Table 3). Hypertension was noted to be associated with a significantly higher number of enhancing primary (β = 1.31, 95% CI 0.78–1.88, *p* < 0.0001) and secondary (β = 1.15, 95% CI 0.29–2.18, *p* < 0.05) vessel segments (Figs. 1 and 2). PACNS was associated with a significantly higher number of enhancing secondary (β = 0.77, 95% CI 0.14–1.39, *p* < 0.05) and tertiary (β = 1.34, 95% CI 0.68–2.01, *p* < 0.0001) vessel segments (Fig. 3). This subgroup analysis indicates that patients with hypertension may have a spatial preference of vessel wall enhancement for the proximal primary and secondary segments, while vessel involvement in PACNS may have a more distal spatial distribution involving the secondary and tertiary segments.

## Discussion

Our results show significant spatial differences in vessel wall enhancement in the intracranial arteries between patients with hypertension, a strong risk factor of ICAD^6^, and PACNS. Hypertension is associated with enhancement of the proximal primary and secondary segments while patients with PACNS showed a distal spatial distribution of vessel wall enhancement. The results from this study are clinically important because most stroke patients have a comorbid diagnosis of hypertension.

Understanding how hypertension may manifest in vessel wall MR imaging studies provides anatomic contextual information to improve image interpretation and achieve higher diagnostic accuracy. The results of this study suggest interpreting the presence of vessel wall enhancement may be more challenging in stroke patients who often have hypertension as a comorbidity.

Possible explanations for vessel wall enhancement in patients with a history of hypertension include increased blood-brain barrier (BBB) permeability with chronically increased systolic blood pressures and the presence of early atherosclerosis. Studies indicate that reactive oxygen species and inflammation are potential mediators of hypertension-induced endothelial cell dysfunction and BBB breakdown^14^. Endothelial cell dysfunction and injury may result in intravenous contrast to leak into the vessel walls manifesting as enhancement. Moreover, early atherosclerotic changes with underlying inflammation^15^ may also manifest as enhancement. A possible explanation for the spatial gradient of vessel wall enhancement associated with hypertension in this investigation may be due to blood pressure gradients throughout the intracranial arterial tree^16^.

Understanding spatial patterns of vessel wall enhancement in the intracranial arteries can improve image interpretation. Our findings show a more distal pattern of enhancement involving secondary and tertiary vessels with PACNS. These findings are concordant with findings from Singhal and colleagues reporting 56% of “middle” arteries (A2, M2, P2, superior cerebellar artery, anterior inferior and posterior inferior cerebellar arteries) and 50% of “smaller distal branches” are abnormal on cerebral angiograms in patients with PACNS^11^.

These results have important implications for clinical practice. First, many stroke patients undergoing VWI have hypertension as a comorbidity, which may confound image interpretation. The spatial distribution of vessel wall enhancement should be taken into account during diagnostic interpretation. Second, some studies suggest the potential use of VWI to monitor treatment response of PACNS to steroids. In patients with a comorbid diagnosis of hypertension and PACNS, understanding that vessel wall enhancement is not isolated to the inflammatory vasculitis is important and can help explain the sometimes variable and long periods of wall enhancement despite clinical improvement during and after treatment. Obusez and colleagues examined 4 patients with PACNS who had follow-up imaging and reported stable enhancement characteristics for a median of 13.5 months^7^. Similarly, another case series reported 4 patients with PACNS follow-up imaging ranging from 2 to 6 months after initiation of immunosuppressive therapy and described stable to reduced enhancement^6^. Neither of these studies described potential comorbid diagnoses that could confound the interpretation of vessel wall enhancement during therapy. Thus, the results of our study suggest that vessel wall enhancement in patients with infarcts due to inflammatory vasculitis may not only have one etiology for wall enhancement, and chronic underlying changes from hypertension should also be considered.

The results from this study also suggest an added diagnostic advantage with imaging the whole brain when an inflammatory CNS vasculitis is suspected. There are technical challenges in image acquisition for vessel wall imaging when attempting to image the whole brain, namely, longer acquisition times and increased vulnerability to motion^17^. However, during clinical image interpretation, assessing for vessel wall enhancement of the tertiary segments is feasible with whole brain imaging and diagnostically helpful for specific indications such as vasculitis. We show that imaging the whole brain and capturing pathology in the distal tertiary vessels provides additional diagnostic information in stroke patients with a clinical suspicion of PACNS. Imaging the whole brain for vasculitis also has the added advantage of identifying parenchymal and leptomeningeal enhancement^18^.

This study has several limitations. First, the sample size is small with only 8 subjects with PACNS, which notably is a rare diagnosis. Second, the external validity of the study is limited with a predominantly North American white population at a single institution. Third, although an analysis with ICAD itself, rather than risk factors, would be informative, histologic confirmation was not possible. Using multicontrast VWI techniques including T2-weighted acquisitions may be helpful but mutlicontrast techniques are limited by longer acquisition times. We acknowledge several technical challenges including the inherent properties of the T1 SPACE acquisition, which can result in T1 shortening of blood on post-contrast imaging. Pre-pulse suppression techniques could be considered to minimize this flow artifact in future investigations. Additionally, our spatial resolution was 0.9 mm. Of note, the imaging endpoint was vessel wall enhancement, which with partial volume averaging, tertiary vessel segment enhancement may be more conspicuous when present. Wall enhancement morphology (e.g., eccentric or circumferential) or wall thickness measurements, which may require a higher spatial resolution was not an imaging endpoint. Finally, larger prospective trials with follow-up VWI exams for longer periods of time and with a larger number of patients with PACNS would also provide more insight into vessel wall enhancement characteristics and changes. The role of vessel wall enhancement, presence of vasa vasorum, and whether these features are an indicator of pre-atherosclerotic states remain unknown. Longitudinal assessments with age-matched healthy controls would be invaluable to establish causality.

In conclusion, we show that a diagnosis of hypertension is significantly associated with a higher number of enhancing vessel wall segments within the proximal and secondary segments. This contrasted with patients with PACNS, who showed a more distal spatial distribution of enhancing vessel wall segments, involving the secondary and tertiary segments. By taking into account spatial distributions of vessel wall enhancement, diagnostic interpretations can be improved to achieve higher accuracy.

## Methods

### Patient selection

In this retrospective study, 100 consecutive patients with VWI from December 2016 to April 2018 were retrospectively identified from a prospectively maintained database at the Massachusetts General Hospital, a tertiary care hospital. The clinical indication for the VWI exams was stroke. Exclusion criteria included repeat follow-up exams, motion degraded exams, age less than 18 years, and diagnoses of nonocclusive vasculopathies such as moyamoya syndrome/disease, reversible cerebral vasoconstriction syndrome (RCVS), and arterial dissection. Demographic data including sex, ethnicity, and cardiovascular risk factors were collected. Medical diagnoses of hypertension, diabetes mellitus, and hyperlipidemia were confirmed from the patient’s medical record with diagnostic criteria established by the treating physician. Diagnoses of PACNS were determined either by a stroke neurologist (GAK) who reviewed the medical record including clinical history, conventional MR imaging (not including the VWI) and using established criteria by Calabrese and Mallek^19^ or by brain biopsy. Our sample size was derived from the cases retrospectively identified during the study period. Ethics approval was obtained from the institutional review board from the Massachusetts General Hospital (IRB#: 2018P000816), which waived the need for informed consent.

### Imaging protocol

Patients were scanned on a 3 T Siemens Skyra or 3 T PRISMA MR scanner (Siemens Healthcare, Erlangen, Germany). A 32-channel head coil was utilized. The VWI imaging protocol included sagittal 3D SPACE T1-weighted sequences (0.9 × 0.9 × 0.9 mm in-plane resolution and slice thickness; repetition time/echo time, 1100/11 ms; FOV 230 × 230; matrix 256 × 256; and time, 7:39 minutes). Post-contrast images were acquired 5 minutes after injection of a gadolinium-containing contrast agent (gadobutrol, Gadovist 1.0 mmol/mL). Coverage included the whole brain. These were reformatted in axial and coronal reformats.

### Image analysis

Two fellowship trained neuroradiologists (JWS and SBR), blinded to the clinical history and radiology reports, reviewed the VWI to qualitatively evaluate for the presence or absence of vessel wall enhancement. Enhancement was determined by comparing pre-gadolinium and post-gadolinium acquisitions. The vessel segments were scored independently by the neuroradiologists. Discordant results were re-reviewed for consensus. An inter-rater reliability analysis was conducted by calculating an unweighted Cohen’s kappa. Twenty-three vessel segments per VWI exam were evaluated: the right and left internal carotid artery termini, A1, A2, A3 segments of the anterior cerebral arteries (ACAs), M1, M2, M3 segments of the middle cerebral arteries (MCAs), P1, P2, P3 segments of the posterior cerebral arteries (PCAs), V4 segments of the vertebral arteries, and the basilar artery^20^. Segments were categorized by branching pattern. A1, M1, P1, internal carotid artery termini, V4, and the basilar artery were categorized as primary segments. A2, M2, and P2 were categorized as secondary segments. A3, M3, and P3 were categorized as tertiary segments. Images were analyzed and interpreted on an Impax 6/0 PACS.

### Statistical analysis

Continuous variables are summarized as mean +/− SD or median +/− interquartile range. Categorical variables are summarized in percentages. Inter-rater reliability is presented by an unweighted Cohen’s kappa. Group comparisons between PACNS and patients with hypertension were done by a Chi square test for categorical variables, logistic regression for binary data, and two-sample t-test for continuous data. Candidate risk factors were selected both based on clinical hypothesis and univariate variable screening. Multivariate Poisson regression models were then fit to examine the association between total counts of enhancing vessel walls in each of the spatial locations (total, primary, secondary and tertiary) and the candidate risk factors. A Poisson regression is a generalized linear model that handles the discrete counts as outcome. It assumed a Poisson distribution conditioning on the covariates and express a log-linear relationship between the mean of the Poisson distribution with the risk factors. The total number of enhancing vessel segments was used as the dependent variable in the univariate analysis. The following 8 predetermined independent variables were included in the model on the basis that these are demographic data and known cardiovascular risk factors of ischemic stroke: demographic information (age, ethnicity, sex), smoking pack years, and clinical diagnoses of hypertension, diabetes mellitus, hyperlipidemia, and PACNS. Risk factors that were significant from the univariate analyses were then included as covariates in the models with dependent variables being the number of enhancing vessels in the primary, secondary and tertiary segments. Multiple comparisons were corrected by controlling for a 5% false discovery rate using the Benjamni-Hochberg procedure. All statistical calculations were conducted with the statistical computing software R (2017, R Foundation for Statistical Computing, Vienna, Austria) and SPSS v19 (Chicago, IL). Two-tailed tests were used with statistical significance defined as p ≤ 0.05. The goodness-of-fit of the models were examined by checking residual plots and the Q-Q plot which compares the residual quartiles with the theoretical quartiles assuming the model is true.

## Supplementary information

Supplementary Tables

## Data Availability

The dataset from the current study are available from the corresponding author upon reasonable request.
