# Supplementary material for: Spatial Distribution of Intracranial Vessel Wall Enhancement in Hypertension and Primary Angiitis of the CNS
Source: Sci Rep. 2019 Dec 17;9:19270. doi: 10.1038/s41598-019-55634-5 (PMC6917817; doi:10.1038/s41598-019-55634-5)
Supplement: Supplementary file 1 — Supplementary Tables [file 41598_2019_55634_MOESM1_ESM.pdf]

Supplemental Material for:

Spatial Distribution of Intracranial Vessel Wall Enhancement in Hypertension and Primary Angiitis of the CNS

Authors: Jae W Song[1], Haochang Shou[2], Emmanuel C Obusez[3], Scott B Raymond[3†], Samuel D Rafla[3], G. Abbas Kharal[4††], Pamela W Schaefer[3], Javier M Romero[3]

Affiliations:
[1]Department of Radiology, Division of Neuroradiology, Hospital of the University of Pennsylvania
[2]Department of Biostatistics, Epidemiology and Informatics, University of Pennsylvania Perelman School of Medicine
[3]Division of Neuroradiology, Massachusetts General Hospital, Harvard Medical School
[4]Department of Neurology, Massachusetts General Hospital, Harvard Medical School

Present positions:
[†]Department of Radiology, University of Vermont College of Medicine
[††] Department of Neurology, Cleveland Clinic

Supplemental Table 1: Inter-rater Agreement

Unweighted Cohen’s kappa (ƙ)	
Internal Carotid Artery Terminus	ƙ=0.39, (95% CI, 0.16-0.61)
Anterior Cerebral Artery	ƙ=0.38, (95% CI, 0.23-0.52)
Middle Cerebral Artery	ƙ=0.45, (95% CI, 0.34-0.55)
Posterior Cerebral Artery	ƙ=0.18, (95% CI, 0.08-0.27)
Vertebral Artery (V4 segment)	ƙ=0.48, (95% CI, 0.35-0.61)
Basilar Artery	ƙ=0.28, (95% CI, 0.004-0.56)

Supplemental Table 2: Median Number of Enhancing Vessel Segments

	All patients	Primary angiitis of the CNS	Hypertension	Primary angiitis of the CNS and Hypertension
All vessel segments	4.0 [1.0, 9.0]	11.0 [3.0, 17.0]	6.0 [3.0, 11]	15 [6.75, 18.25]
Primary#	2.0 [0, 6.0]	6 [0.5, 9.75]	5 [0.5, 7.0]	8.00 [3.75, 10.25]
Secondary**	0 [0, 2.0]	2.5 [0.25, 4.75]	1.0 [0, 3.0]	4.00 [0.75, 5.00]
Tertiary††	0 [0, 1.0]	3.00 [1.25, 3.75]	0 [0, 1.5]	3.00 [0.75, 4.50]

*Presented as median and interquartile ranges [IQR]
Basilar artery and bilateral A1, M1, P1, V4, ICA terminus (11 segments)
**Bilateral A2, M2, P2 (6 segments)
†† Bilateral A3, M3, P3 (6 segments)
